# Deep visual domain adaptation and semi-supervised segmentation for understanding wave elevation using wave flume video images

**DOI:** 10.1038/s41598-021-01157-x

**Published:** 2021-11-05

**Authors:** Jinah Kim, Taekyung Kim, Sang-Ho Oh, Kideok Do, Joon-Gyu Ryu, Jaeil Kim

**Affiliations:** 1 Coastal Disaster Research Center, Korea Institute of Ocean Science and Technology, Busan, 49111 South Korea; 2 Department of Civil Engineering, Changwon National University, Changwon-si, 51140 South Korea; 3 Department of Ocean Engineering, Korea Maritime and Ocean University, Busan, 49111 South Korea; 4 Satellite Wide-Area Infra Research Section, Electronics and Telecommunications Research Institute, Daejeon, 34129 South Korea; 5 School of Computer Science and Engineering, Kyungpook National University, Daegu, 41566 South Korea

**Keywords:** Physical oceanography, Computer science

## Abstract

Accurate water surface elevation estimation is essential for understanding nearshore processes, but it is still challenging due to limitations in measuring water level using in-situ acoustic sensors. This paper presents a vision-based water surface elevation estimation approach using multi-view datasets. Specifically, we propose a visual domain adaptation method to build a water level estimator in spite of a situation in which ocean wave height cannot be measured directly. We also implemented a semi-supervised approach to extract wave height information from long-term sequences of wave height observations with minimal supervision. We performed wave flume experiments in a hydraulic laboratory with two cameras with side and top viewpoints to validate the effectiveness of our approach. The performance of the proposed models were evaluated by comparing the estimated time series of water elevation with the ground-truth wave gauge data at three locations along the wave flume. The estimated time series were in good agreement within the averaged correlation coefficient of 0.98 and 0.90 on the measurement and 0.95 and 0.85 on the estimation for regular and irregular waves, respectively.

## Introduction

Coastal observation using remote sensing and unmanned systems has led to advances in understanding and modeling nearshore processes, such as shorelines, surf zones, and inner shelves, by allowing long-term observation facilities in coastal areas. In particular, land-based remote sensing devices, such as shore-based camera and video systems, enable synoptic surface and subsurface observations with high temporal resolutions over long time scales, even in the case of extreme events^1^. These devices have also been used to measure shoreline positions and infer subsurface morphology as well as to measure the water waves of the inner surf and swash, in addition to sub-aerial bathymetry^2–4^.

As well as field experiments, laboratory studies should be a component of investigations of nearshore phenomena. Experiments with wave flumes that satisfy scaling laws enable controlled experiments on some nearshore processes, which can yield a variety of data on the parameterization of specific processes. Moreover, laboratory environments can also be useful in evaluating the performance of video systems as a measurement tool. Video systems allow the easy and efficient acquisition of large amounts of data that are highly dense in terms of time and space, in both field and laboratory experiments for investigations; however, their applicability depends on the accuracy, reliability, and robustness of processes that can be visually recorded by optical sensors as real physical quantities, compared to in-situ acoustic sensors. Moreover, vision-based methods are widely used in place of, or in parallel with, conventional measurements, and must be supported by the generalization of analysis methodology, ease of application to other data sets, the robustness of environmental conditions, and the economic efficiency of imaging tools and processing machines.

Modern computer vision technology, based on artificial intelligence has evolved dramatically across a wide range of vision applications, such as image classification, facial recognition, object identification, and video analysis in robots and autonomous vehicles. In particular, deep learning-based methods have given way to a new generation of image segmentation models^5^ at a hitherto inconceivable level and with remarkable performance improvements, often achieving a high level of accuracy in perceiving precisely real-world objects, which has resulted in a paradigm shift in the field.

The success of deep learning has been driven by the emergence of large scale training data, which renders multi-domain learning an interesting challenge. In computer vision, a domain often refers to a dataset in which samples correspond to the same underlying data distribution. It is common for multiple datasets with different data distributions to be proposed to target the same or similar problems, for example the person re-ID problem^6^. Combining heterogeneous datasets together can diversify the training data, thus making the learned features more robust. However, annotating labeled datasets for new tasks in different domains is extremely expensive and time-consuming processes, and sufficient training data may not always be available. Fortunately, the big data era has made a large amount of data available for other domains and tasks. Domain adaptation is the capacity to apply an algorithm trained in one or more source domains to a different but related target domain^7^. Multi-domain learning seeks to resolve this problem by simultaneously utilizing datasets from different domains for domain adaptation.

Consequently, we propose two approaches to water elevation measurement from side-view video images and estimation from top-view video images through deep image segmentation and deep visual domain adaptation, respectively, using time-synchronized, multi-view video images that were acquired via wave flume experiments. We employ video images collected under laboratory conditions for semi-supervised image segmentation^8^ to measure water elevation from a side-view perspective and evaluate performance using wave gauge data. We also employ multi-view laboratory video images from a secondary camera for deep visual domain adaptation to estimate water levels from top-view videos and to enable the generalization of the methodology and its applicability to other datasets.

## Results

### Data

**Figure 1:**
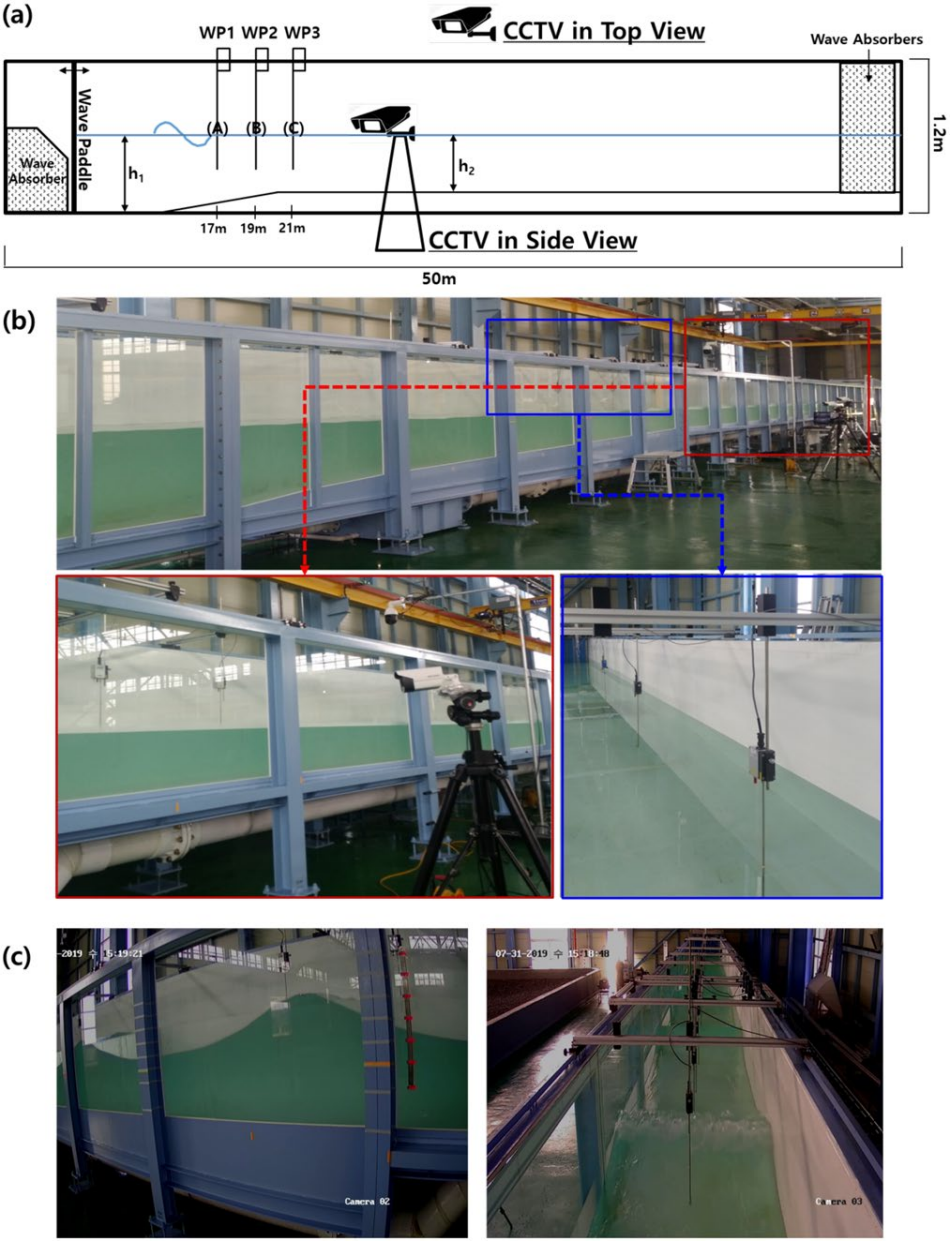
(**a**) The illustrated experimental setup in (**b**) 2D wave flume. Three capacitance wave gauges (WP1, WP2, and WP3) and two CCTVs in side and top view are installed and (**c**) the samples in side and top view on from left and right.

The experiments reported herein were carried out to acquire video data at the Physical Experiment Building 2D wave flume at the Korea Institute of Ocean Science and Technology^9^. Figure 1 illustrates the general layout of the flume, including the placement of the capacitance probes and video cameras. The flume is 50 m long, 1.2 m wide, and 1.6 m high. A schematic diagram of the plan and side views of the wave flume is shown in Supplementary Fig. 3. The AwaSys software system^10^ is incorporated to control the drive signal for the wave paddle. The starting and ending locations of the slope are 10 and 20 m, respectively and the angle of the slope is 0.035.

Wave were generated by piston-type wave paddle that moved laterally to produce either regular or irregular waves. Three capacitance-type wave gauges were installed to measure the water surface elevation at three locations along the flume (WP1, WP2, and WP3 in Fig. 1) and the data was collected at a frequency of 50 Hz.

As described in Table 1, various experiments were performed by generating both regular and irregular waves to obtain videos with conditions that are actually able to occur in coastal areas. JONSWAP spectrum was applied to generate irregular waves to simulate a random sea state. A total of 57 video datasets were attained, and the length of each run varied from 5 to 20 min depending on the experimental conditions.

**Table 1:** Experimental conditions for regular and irregular waves in the wave flume.

Wave type	Water depth (cm, $h_2$)	Wave period (s)	Wave height (cm)	Number of run
Regular	40	$T=1.50$–2.50 (0.25 interval)	$H=20$–35 (5 interval)	2
	50			5
	60			20
Irregular	50	$T_s=1.50$–2.50 (0.25 interval)	$H_s =15$–25 (5 interval)	12

The experiment was conducted with three different water depth conditions of 40, 50, and 60 cm. Here, the water depth refers to $h_2$ in Fig. 1a. In the case of regular waves, the wave period (*T*) ranged from 1.5 to 2.5 s with and interval of 0.25 s, whereas the wave height (*H*) varied from 20 to 35 cm at an interval of 5 cm for each wave period. In the case of irregular waves, the conditions of significant wave height ($H_s$) and significant wave period ($T_s$) were the same as regular waves. These wave conditions correspond to the conditions specified in the wave generator. Experimental conditions are more described in Supplementary Tables 1 and 2.

A closed-circuit television (CCTV) camera with a temporal resolution of 30 frames per second (fps) and spatial resolution of $1920\times 1080$ was used to record a series of experimental scenes with two different viewpoints from the side and top of the wave flume (see Fig. 1a).

For the geo-rectification, perspective projection transformation^11^ was performed using fixed wave flume frames at 2 m intervals and stickers for grids separated by 100 cm as a reference point (landmark) as shown on the left of Fig. 1c. The transformation matrix for perspective projection was calculated using landmarks for each fixed wave flume frame at 2 m intervals, and the three wave gauges locations (WP1, WP2, and WP3 in Fig. 1a) to be used as ground-truth were mapped to the geo-rectified image. Through this image pro-processing process, the spatial resolution of the original video image is downscaled to $620\times 220$, and the real distance per pixel is 0.5 cm.

### Evaluation metrics

The Dice coefficient (Dice)^12^ was used to evaluate the performance of the water region segmentation model for the time-stack images. In this accuracy metric, we compared the ground-truth mask($S_g$) with the segmentation map ($S_e$) inferred from the U-Net-based segmentation model in the test dataset. The Dice coefficient is a measure of the overlap between the two masks and ranges from 0 ~ 1, where a 1 denotes a perfect and complete overlap:

1 $$\begin{aligned} Dice(S_g, S_e) = \frac{2|S_g \bigcap S_e|}{|S_g|+|S_e|} \end{aligned}$$

The root-mean-square-error (*RMSE*) and Pearson correlation coefficient (*R*) were also used to evaluate the performance of the water level measurement using side-view images in the semi-supervised water level segmentation mode and the water level was estimated from the top-view video images using deep visual domain adaptation. $x_i$ is the observation of the water level using the wave gauge data regarded as ground-truth. $y_i$ is the measured water elevation in image segmentation through the side-view video image, and the estimated water elevation in visual domain adaptation through the top-view video image.

2 $$\begin{aligned} RMSE(X,Y)= & {} \sqrt{\frac{1}{N}\sum _{t=1}^{N}(x_i - y_i)^2} \end{aligned}$$

3 $$\begin{aligned} R (X,Y)= & {} \frac{{}\sum _{i=1}^{n} (x_i - {\overline{x}})(y_i - {\overline{y}})}{\sqrt{\sum _{i=1}^{n} (x_i - {\overline{x}})^2(y_i - {\overline{y}})^2}} \end{aligned}$$

### Experimental results

For evaluating the accuracy of the automatically-produced segmentation mask using the *FloodFill* algorithm, the Dice coefficient was calculated on the basis of the valid slice images passed to the Supplementary Alg. 1, which are shown in Table 2. In the case of regular waves, on average, about 95 % of the pixels were determined to match in the entire slice image at three locations, i.e. the Dice coefficient Table 2. However, for irregular wave image, the averaged pixel matching rate at three locations was about 85 %. There may be several reasons why average segmentation accuracy from the three locations for irregular wave is lower than for regular wave, but the most common cause is that the waveform itself is likely to have more undulations for irregular waves even if the average wave slope is the same as regular waves. Also, in the case of irregular waves, an afterimage effect occasionally occurs in the time-stack image due to wave transformation such as transient wave breaking, which reduces the clarity of the boundary between the background and the water. Furthermore, as a result of examining the accuracies at three different locations individually for regular and irregular waves, it was found that the closer to the camera, the higher segmentation accuracy. It is believed that the closer the distance from the camera, the higher quality of the image, which helps the segmentation algorithm to more accurately distinguish the background form the waves.

**Table 2:** Accuracy of segmentation mask produced by *FloodFill* algorithm for automatic labeling data at (A), (B), and (C) locations.

	Wave type	(A)	(B)	(C)
Total number of slice image	Regular	251	251	251
	Irregular	335	335	335
Number of valid slice image	Regular	91	50	75
	Irregular	247	162	61
Dice coefficient	Regular	0.93	0.96	0.97
	Irregular	0.78	0.84	0.92

**Figure 2:**
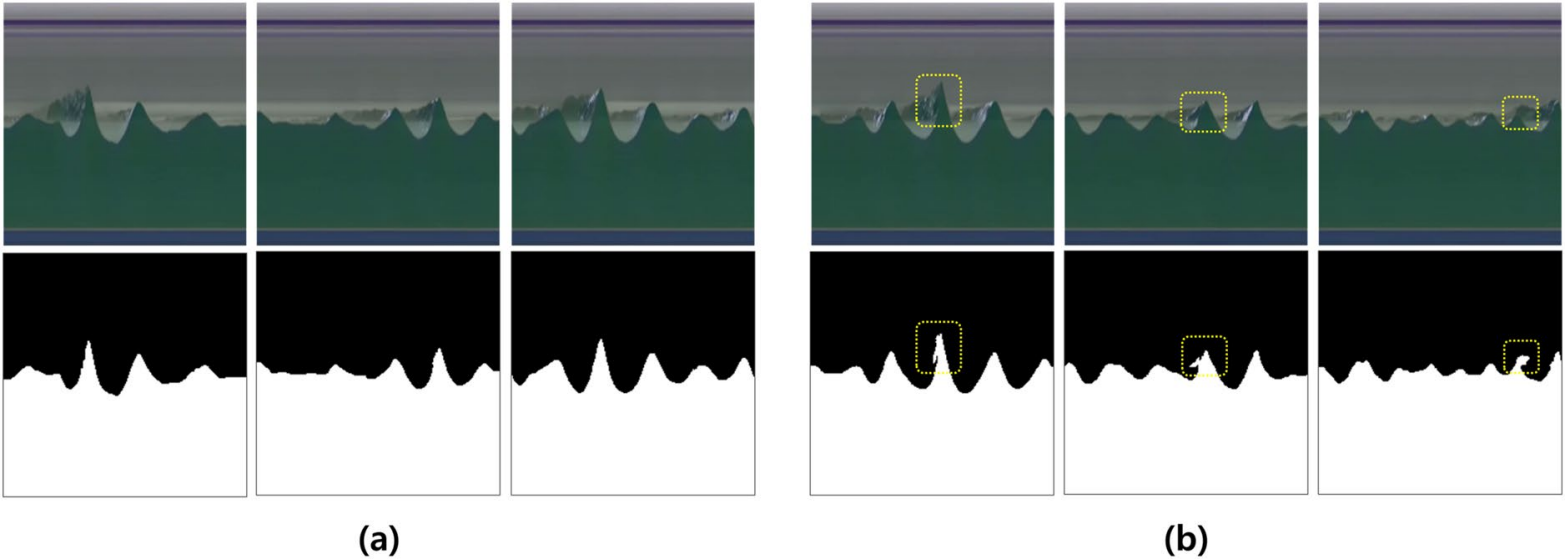
Samples of (**a**) robust and accurate segmentation results and (**b**) inaccurate results against noise due to the wave breaking and afterimage effect of propagating waves.

Figure 2 shows the results of a robustly- and accurately- segmented map (Fig. 2a) for noise generated by the wave breaking and afterimage effect, caused by the propagating waves. However, the yellow square in the Fig. 2b shows a map that is partially inaccurately segmented due to the wave breaking and afterimage effect.

**Figure 3:**
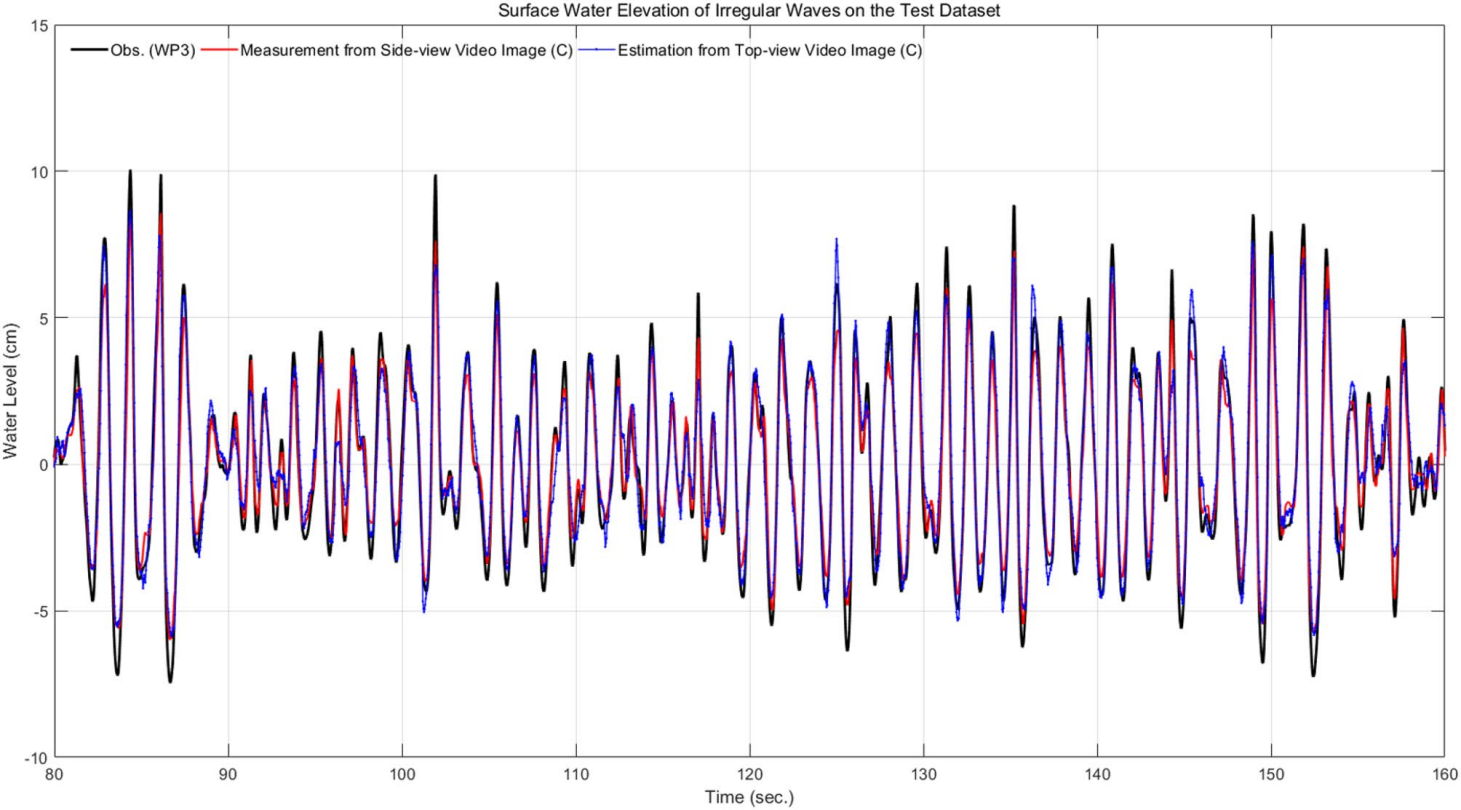
Time series of measured (red line) and estimated (blue dotted line) water elevation from side-view and top-view video images, respectively at the location of (C) with ground-truth (black line) from wave gauge data at the location of WP3 for the test data of the irregular wave experimental video.

Figure 3 shows the time series of the measured water level from the side-view and estimated water level from the top-view images in the form of semi-supervised image segmentation and deep visual domain adaptation, respectively at location (C), with the wave gauge data at the corresponding location of WP3.

**Figure 4:**
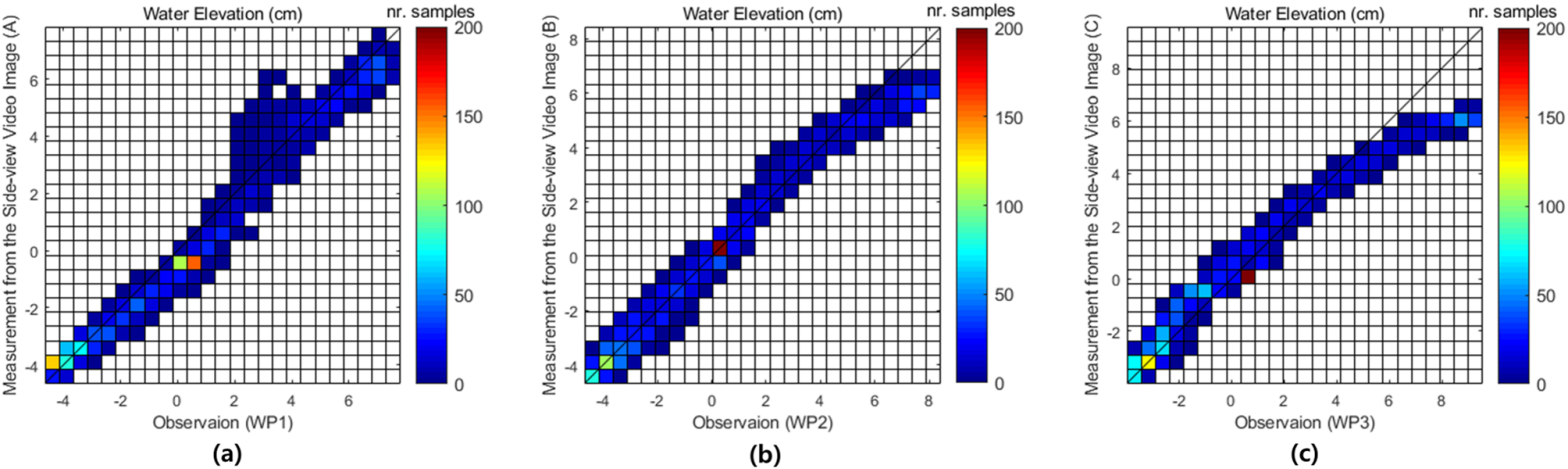
Density scatter of observation of water level using wave gauges at three locations of WP1, WP2, and WP3 and measurement of water elevation from side-view video images using the semi-supervised image segmentation network at the location of (A), (B), and (C) for the regular wave experimental case with $h_1=75.4~\hbox {cm}$, $h_2 =40~\hbox {cm}$, $T=2.25~\hbox {s}$, and $H=10~\hbox {cm}$.

**Figure 5:**
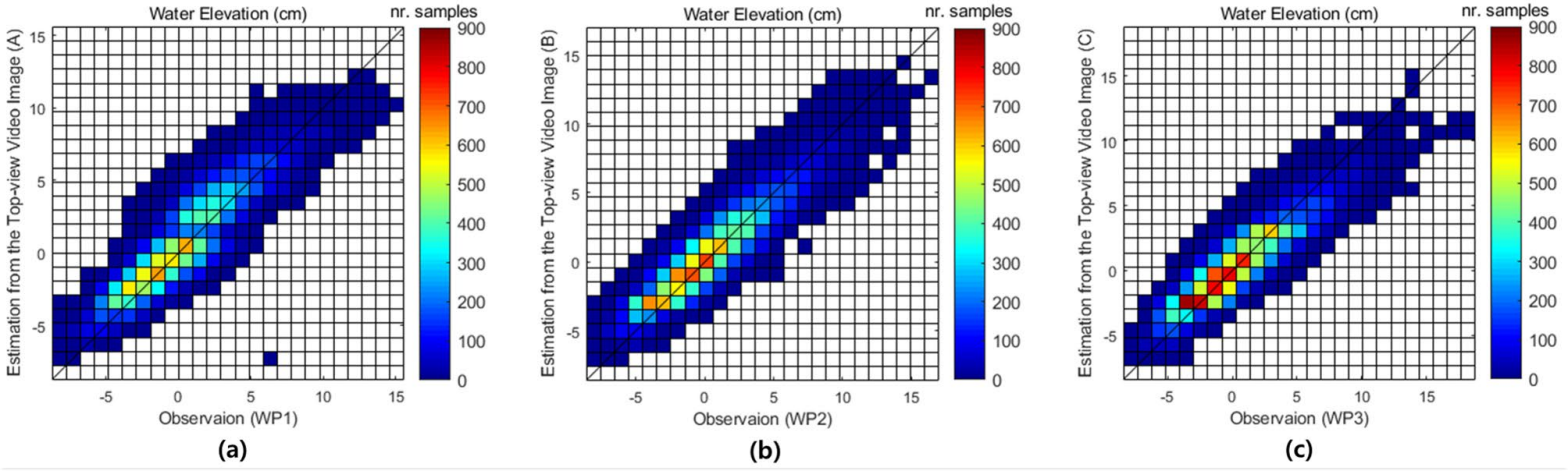
Density scatter of observation of water level using wave gauges at three locations of WP1, WP2, and WP3 and estimation of water elevation from top-view video images using the deep visual domain adaptation network for the irregular experimental case with $T_s =2.5~\hbox {s}$. and $H_s =15~\hbox {cm}$.

Figures 4 and 5 show density scatter plots with observation and measured and estimated water level for regular and irregular wave experimental cases in the test datasets, respectively at the three location of WP1-(A), WP2-(B), and WP3-(C). The Figs. 4 and 5, the water elevations measured and estimated at (A), (B), and (C) are values obtained from the side-view video images and top-view video images, respectively. The wave condition for the regular wave in Fig. 4 is the case where $h_1 =75.4~\hbox {cm}$, $h_2 =40~\hbox {cm}$, $T= 2.25~\hbox {s}$, and $H=10~\hbox {cm}$ (Supplementary Table 1). The correlation coefficient *R* of the WP1-(A), WP2-(B), and WP3-(C) datasets for regular wave are 0.97, 0.90, and 0.81, respectively. And the wave condition for the irregular wave in Fig. 5 is the case where $T_s=2.5~\hbox {s}$ and $H_s =15~\hbox {cm}$ (Supplementary Table 2). The correlation coefficient *R* of the WP1-(A), WP2-(B), and WP3-(C) datasets for irregular wave are 0.93, 0.88, and 0.83, respectively. In both figures, the tendency to underestimate when the surface water elevation is high can be see. The tendency of underestimation was similarly repeated as the absolute water level increased, regardless of the location of (A), (B), and (C) in other regular and irregular waves’ test results. This will be improved if the training data in the case of high water elevation is additionally used for model training.

**Table 3:** Performance of measured water elevation from side-view video images through semi-supervised image segmentation on test dataset comparing with wave gauge data as a ground-truth.

Wave type	*R*			*RMSE* (cm)		
	(A)	(B)	(C)	(A)	(B)	(C)
Regular	0.96	0.98	0.99	3.27	2.25	2.39
Irregular	0.83	0.89	0.98	2.37	1.94	1.08

Table 3 shows the performance of the water level segmentation compared to the wave gauge data at three locations using the evaluation metrics of the correlation coefficient *R* and *RMSE* at the three locations of (A), (B), and (C), respectively. The average *R* and *RMSE* values were 0.98, 0.90, 2.6, and 1.80, respectively, for the regular and irregular wave experimental video images in the test data. In the case of *R*, the irregular waves exhibited a slightly lower correlation than the regular ones, but the irregular wave error was lower in the case of *RMSE*.

In order to evaluate the performance of the water level estimation by means of joint representation learning through deep visual domain adaptation, the metrics of the *R* and *RMSE* were computed in Table 4. The average of *R* and *RMSE* values were 0.95, 0.85, 4.13, and 2.28, respectively, for the regular and irregular wave experimental video images of the test data on deep visual domain adaptation.

**Table 4:** Performance of estimated water elevation from top-view video images through deep visual domain adaptation on test dataset comparing with wave gauge data as a ground-truth.

Wave type	*R*			*RMSE* (cm)		
	(A)	(B)	(C)	(A)	(B)	(C)
Regular	0.93	0.96	0.97	4.87	3.59	3.93
Irregular	0.78	0.84	0.92	2.75	2.31	1.77

By mapping the top-view video images to the corresponding side-view ones, the performance of the estimated water elevation was slightly lower than the water level measured in the latter, but the time series shown in Fig. 3 shows the performance that can be utilized as a water elevation estimate within a sufficiently acceptable error range.

**Table 5:** Averaged performance of waver surface elevation measurement and estimation of propagating water waves with semi-supervised image segmentation on side-view image and deep visual adaptation on top-view image, respectively in test dataset.

Evaluation metric	Wave type	Measurement (side-view video images)	Estimation (top-view video images)
Averaged *R*	Regular	0.98	0.95
	Irregular	0.90	0.85
Averaged *RMSE* (cm)	Regular	2.64	4.13
	Irregular	1.80	2.28

The results of water elevation measurement from the side-view video images and estimation from the top-view video images were in good agreement compared to the ground-truth wave gauge data in three locations, with averaged correlation coefficient *R* of 0.98 and 0.95, and *RMSE* of 2.6 and 4.13, respectively, for regular waves as shown in Table 5. In addition, the averaged *R* and *RMSE* for the measurement and estimation of the water elevation for irregular waves are 0.90, 0.95, 1.80, and 2.28, respectively.

For water elevation measurement from the side-view video images and estimation from the top-view video images, the reason (C) has the highest accuracy, and the next (B), and (A) has the lowest accuracy seems to be because the location of the camera that record the video is closet to the (C) location. As shown in Fig. 1a, the intervals of (A), (B), and (C) are each 2 m apart, and the cameras are closest to the location (C). In Table 2, the Dice coefficient for automatically labeled segmentation mask of each location is shown in the same order of above segmentation result.

**Figure 6:**
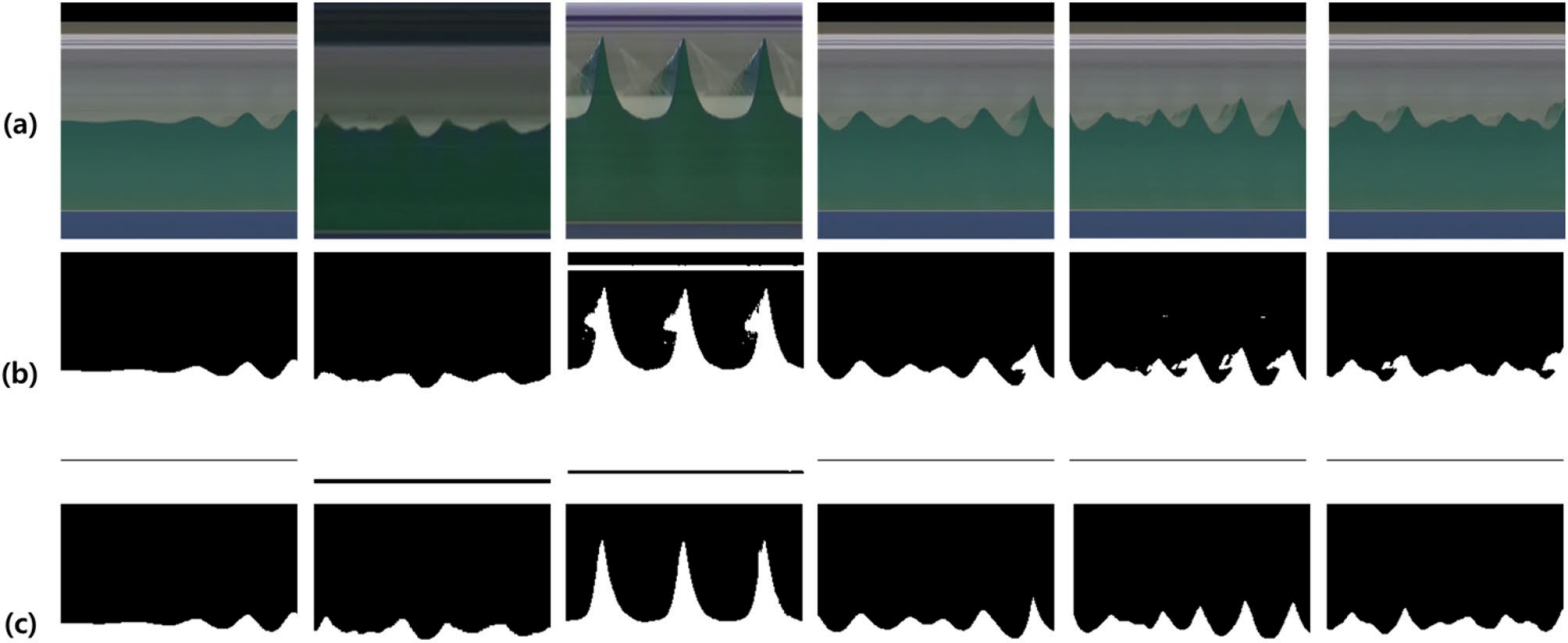
Samples of segmentation results in test dataset (**a**) time-stack image, i.e., input image and (**b**) segmented result by applying slope difference distribution based threshold selection method, and (**c**) segmented result by applying the proposed U-Net based image segmentation network described in Supplementary Fig. 2.

**Table 6:** Comparison of segmentation performance using Dice coefficient on time-stack images. Intensity-based method is a slop difference distribution based segmentation, and the semi-supervised U-Net is the proposed method descried in Supplementary Fig. 2.

Wave type	Intensity-based segmentation			Semi-supervised U-Net		
	(A)	(B)	(C)	(A)	(B)	(C)
Regular	0.8997	0.9273	0.9694	0.9992	0.9994	0.9996
Irregular	0.8588	0.8856	0.9672	0.9985	0.9994	9997

To evaluate the effectiveness of the semi-supervised learning method for the time-stack image segmentation, we compared the proposed semi-supervised method with an intensity based segmentation method using slope difference distribution for threshold selection^13^. Table 6 describes the segmentation methods by the two methods in terms of Dice coefficient with the ground-truth segmentation maps. Figure 6 shows the samples of the segmentation results. For the regular type of waves, the two methods achieved comparable results on the segmentation task. However, for the irregular type of waves where we can observe various photometric changes by afterimage effect of propagating waves and occasionally appearing wave breaking in time-stack images, the learning-based method provided more robust segmentation maps enough to measure the surface water elevations. The experimental results indicate that learning the complex patterns of waves in the time-stack images helps the water level measurement despite of large variations in waves and observation environments.

## Discussion and conclusion

In coastal research, video image-based remote sensing allows continuous, area-based, and high-resolution spatio-temporal monitoring. Furthermore, such data, with its high rates of irregularities and nonlinearities, enables data-driven modeling based on deep learning technology. This can lead to major progress, especially in the understanding of nearshore coastal processes.

Water elevation is the most basic physical quantity to be extracted using coastal video images. In this study, we propose a deep image segmentation method via semi-supervised learning to alleviate the labor-intensive data labeling issue in building training dataset. Furthermore, given that it is not easy to obtain a cross-sectional (side-view video images) that facilitates the measurement of height in real coastal areas, a deep visual domain adaptation procedure that can estimate height via top-view video images is also proposed. In coastal areas, it is necessary to use data from different sources (modalities), such as sensory, radar, satellite, and numerical simulations, instead of cross-sectional video imagery.

In this study, we propose two approaches to water elevation measurement from the side-view video images and estimation from the top-view video images through deep image segmentation via semi-supervised learning and deep visual domain adaptation, respectively using time-synchronized, multi-view video images that ere acquire via wave flume experiments. Two tasks for measuring and estimating water elevation on the basis of side- and top-view video images were applied to experimental videos with propagating regular and irregular water waves in a 2D wave flume via the semi-supervised image segmentation based on U-Net architecture and the deep domain adaptation method.

The water level measurement from the side-view video images and estimation from the top-view video images results were in good agreement compared to the ground-truth wave gauges in three locations, with average correlation coefficients *R* of 0.98 and 0.90 on the measurement and 0.95 and 0.85 on the estimation for regular and irregular waves, respectively. Moreover, the averaged RMSE (cm) is 2.64 and 1.08 on the measurement and 4.13 and 2.28 on the estimation for regular and irregular waves, respectively.

In terms of performance, the main cause of the error to measure water elevation from side-view video image is that the boundary between the background and the wave is not completely separated in the time-stack image due to afterimage effect of propagating waves and occasionally appearing wave breaking, as shown in Fig. 2. Therefore, the water elevation estimation error through the top-view video image includes both the error for domain adaptation learning and the wave elevation measurement error through the image segmentation using the side-view video images (See Tables 3 and 4).

The following conclusions can be described from Tables 2, 3 and 4 for image segmentation, water level measurement, and water level estimation, respectively. The location closest to the camera had the best image quality, so it was the best in performance for the image segmentation (spatial separation of background and water), and the water level measurement from the segmented images, and the water level estimation. In addition, it was possible to obtain the most robust and excellent image segmentation, water level measurement, and water level estimation results at the location closest to the camera despite various photometric changes owing to the afterimage effect of propagating waves and occasionally appearing wave breaking. But, (C), which is the closest location to the camera, has the shallower water depth compared to other locations of (A) and (B), which may degrade performance under steeper wave conditions. However, from the results in Tables 2, 3, and 4, it can be seen that even under these conditions, a good quality image at the location closest to the camera show the highest performance. This shows that the proposed learning-based methodology to understand the propagating process of waves can maintain good performance in water elevation measurement and estimation even under physical conditions that degrade performance if only high-quality images can be obtained. It is natural, but the best way to increase the accuracy when acquiring physical quantities through images is to place the camera close to the object to be quantified.

The proposed semi-supervised learning and domain adaptation approaches maximize their usability and applicability to video-based remote sensing studies that make use of deep learning technology. Furthermore, the proposed deep visual domain adaptation method achieves more flexible scheme with heterogeneous modalities, because it allows more domain-specific feature extractions to be learned and maps the learned features to a common space within which the domains are close to one another while maintaining good task performance.

The proposed learning-based approach is more robust to afterimage effect caused by the propagating wave and occasionally appearing wave breaking the external environmental conditions than the conventional intensity-based segmentation method and has high generalization and applicability with improved performance, and this is a promising approach for the study of nearshore coastal wave processes. Although it is a method that can estimate the sea surface elevation from the top-view video images and is highly applicable to the coastal area, a varying wave incidence angle, multi-directional seas, breaking waves, natural lighting condition, location of camera must be considered in the real world domain. It is expected that deep learning-based coastal video enhancement and hydrodynamic scene separation algorithms such as upsampling can be used in the image pre-processing step by collecting enough field data for resolution degradation due to camera position or large variability in natural light except at night^4,14^. In addition, even in the present 2D wave flume experiment, some errors in water elevation estimation occurs due to the afterimage effect caused by wave breaking (see Fig. 2b). In order to improve this, it is necessary to obtain more diverse conditions experiments and videos on breaking wave intensively and use them as learning data. Furthermore, for various wave incidence angles and multi-directional seas such as the real coast, it is essential to obtain sufficient learning data through a three-dimensional (3D) wave flume experiment before field application to ensure the water level estimation performance. 3D wave flume experiment for this is being planned for the next study. In the future, the findings of this study can be expanded upon in the direction of modeling the entire water evolution process.

## Methods

To quantify the water level in the multi-view wave flume videos, two tasks had to be performed on the video images obtained from the two cameras: continuous water level measurement from the side-view video images and water level estimation from the top-view video images. To measure the water level in the side-view video images and estimate that in the top-view video images, semi-supervised learning for image segmentation and deep visual domain adaptation techniques with multiple Convolutional Neural Networks (CNNs) were developed, respectively.

First, the image segmentation^5^ in computer vision is typically first used to delineate object or region boundaries in images. In deep image segmentation approaches, it is usually necessary to provide a paired dataset with images and corresponding ground-truth masks for the fully-supervised learning of segmentation networks. However, the preparation of training data is a time-consuming and labor-intensive task, especially for video datasets. In this study, we implemented a semi-supervised learning for segmentation-based water level measurements from the side-view video images.

Semi-supervised learning^15^ is a type of machine learning that employs a combination of a small amount of labeled data (supervised learning) and a large amount of unlabeled data (unsupervised learning) to train neural networks while avoiding the challenges of processing a large amount of labeled training data. The approach enables us to train a segmentation network with both unlabeled and labeled samples, which are typically assumed to have been sampled from the same or a similar distribution.

Next, deep domain adaptation^7^ is a technique that learns transferable representations that disentangle the exploratory factors of variations underlying the data samples and group features so that a specific trained model can be used directly for the other tasks of different domain divergences (i.e., distribution shifts or feature space differences). In other words, the domain adaptation utilizes the labeled data in one or more relevant source domains to execute new tasks within a target domain. The goal is to identify or construct a common representation space for the two domains.

In our approach, we implemented a domain adaptation technique to map the high-level features of the top-view video images to those of the side-view video images, which were learned for the water level estimation on the basis of the labels. In the following sections, we describe the semi-supervised segmentation and domain adaptation methods employed.

**Figure 7:**
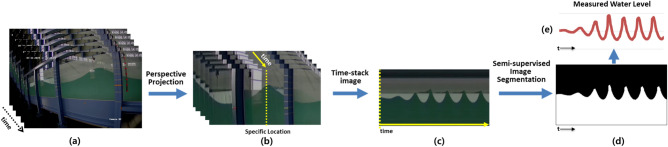
Procedure of water level measurement in the side view video images based on semi-supervised image segmentation: (**a**) consecutive image of side view video, (**b**) projected consecutive image by geo-rectification, (**c**) time-stack image extracted from one specific location, (**d**) segmented time-stack image produced by semi-supervised image segmentation for water region, and (**e**) measured water level from the segmented time-stack image (**d**).

### Semi-supervised image segmentation for measuring water elevation from side-view video image

The procedure for image segmentation is shown in Fig. 7 for the measuring of surface water elevation in wave flume video images. Figure 7a,b show consecutive images extracted from the side-view wave flume video and geo-rectified consecutive images in the region of interest, respectively. Figure 7c presents a time-stack image with the time cited at the target location (*y*-axis) and cross-section position on the time (*x* axis). The time-stack image is a time series of optical intensity from an array of pixels at any desired location in the images. Therefore, the vertical yellow dotted line in Fig. 7b becomes the desired location (*y*-axis in Fig. 7c), and if consecutive images of video are stacked in the *x*-axis) direction along this dotted line, the time-stack image of the corresponding location is made as in Fig. 7c. That is, the vertical axis of Fig. 7c is the desired location indicated by the yellow dotted line in Fig. 7b, and the yellow solid line on the horizontal axis indicates time. Using semi-supervised learning from the time-stack image, the boundary of the water is determined as shown in Fig. 7d. Based on this segmented images, the water level can be measured as shown in Fig. 7e.

In the case of semi-supervised learning, the *FloodFill* algorithm^16^ is applied to automatically produce a small amount of labeled data. *FloodFill* is a region-growing method that generates region masks of target objects based on the brightness and color difference between the reference pixel and adjacent pixels in the input image. However, it is difficult to set thresholds for brightness and color differences, and the algorithm is sensitive to large changes in pixel intensities and the shape of target objects. Therefore, after applying *FloodFill* to the time-stack images, only part of the valid region by the evaluation process (Supplementary Alg. 1) was used as the labeling data, as shown in Supplementary Fig. 1.

With the small number of valid segmentation maps, we first train a segmentation network based on the U-Net^17^. The segmentation network illustrated in Supplementary Fig. 2 performs image segmentation by learning the features of regions corresponding to the water in the time-stack images in order to distinguish between the propagated water waves and background in the experimental videos. The proposed U-Net architecture (Supplementary Fig. 2) comprises two parts, namely an encoder that learns the latent feature of time-stack images from raw video images to capture the context, and a decoder that restores the regions of water that enables precise localization to be performed. The skip connection in the architecture concatenates the output features of the *Encoder Block* to those of the *Decoder Block* in order to improve the segmentation quality by preserving local details on the wave surface.

Let the U-Net model parameterized by $\theta$ be $U(I_{stack};~\theta )$ where $I_{stack}$ is an input slice from the time-stack images. The model receives a set of slice images $220\times 220 \times 3$ in size and outputs a probability map with the same size in which each element has a value range of [0, 1]. In the output map, the threshold is applied based on 0.5 to obtain a segmentation map with a value of 1 for the water region and 0 for the background region. In order to train the network, we use a pixel-wise binary cross-entropy loss between a given input slice image and the corresponding segmentation map *m* as follows:

4 $$\begin{aligned} \begin{aligned} L_{seg}=-\sum _{x \in I_{stack}} w(x) \log (P_{U}(x)) + (1-w(x)) \log (1-P_{U}(x)) \end{aligned} \end{aligned}$$

where *x* is a pixel of the input slice and *w* indicates the background ($w(x)=0$) and water region labels ($w(x)=1$) in the segmentation map ($m_{stack, n}$). In turn, $P_{U}(x)$ is the pixel-wise probability from the Sigmoid function in the segmentation network. Furthermore, we acquired the slice images and corresponding valid segmentation maps of $220\times 220$ window size by sliding the slice window from the left to the right with random steps for the data augmentation (Supplementary Fig. 1).

In order to improve the robustness of the segmentation network, we implement a semi-supervised learning method as described in Supplementary Alg. 2. The flow chart of the semi-supervised learning method is depicted in Supplementary Fig. 4. As the ground-truth (valid) segmentation maps for all slice images are not initially given, we first segment the slice images without valid segmentation maps using the segmentation network, which was trained using only the initial labeled data. Next, we perform validity evaluation using Supplementary Alg. 1 to identify correctly-segmented image slices within the segmentation network. Then, we again train the segmentation networks using the valid segmentation maps, which are evaluated as a pseudo-labeled dataset. This semi-supervised learning process is repeated a given number of times. The water level at each time point is then determined by measuring the wave height from the bottom to the top (water surface) of the water regions in the time-stack images.

### Deep domain adaptation for estimating water elevation from top-view video image

**Figure 8:**
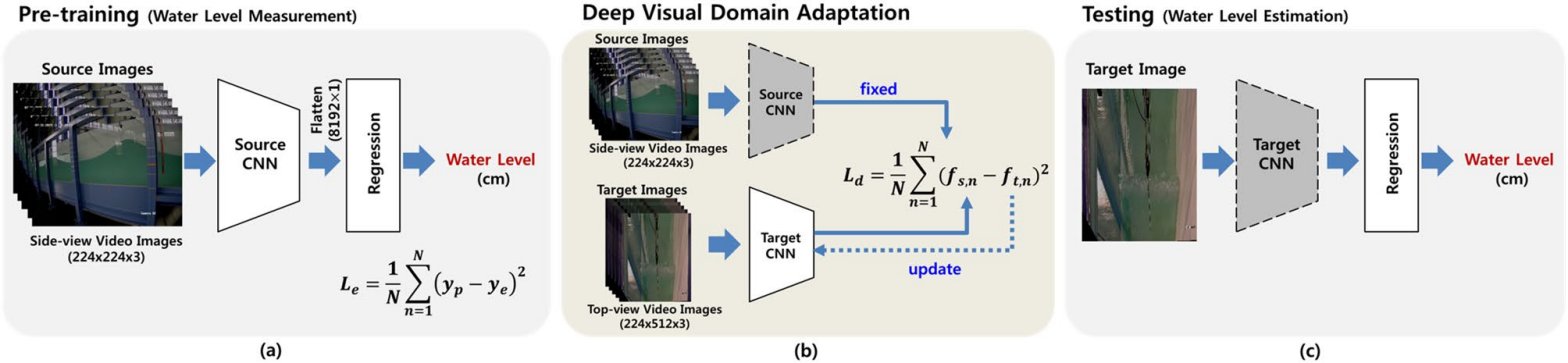
Procedure of domain adaptation (**a**) pre-training of **Source CNN** for water level measurement, (**b**) deep visual domain adaptation for training **Target CNN**, and (**c**) regression model for testing to estimate water level from top-view video images.

As is shown in Fig. 8, the deep visual domain adaptation achieves independent source and target domain mapping by untying the weights, and the parameters of the target model were initialized using a pre-trained source. In the proposed approach, domain adaptation maintains good performance in the source task while at the same time obtaining a joint representation close to each other for the target task.

**Figure 9:**
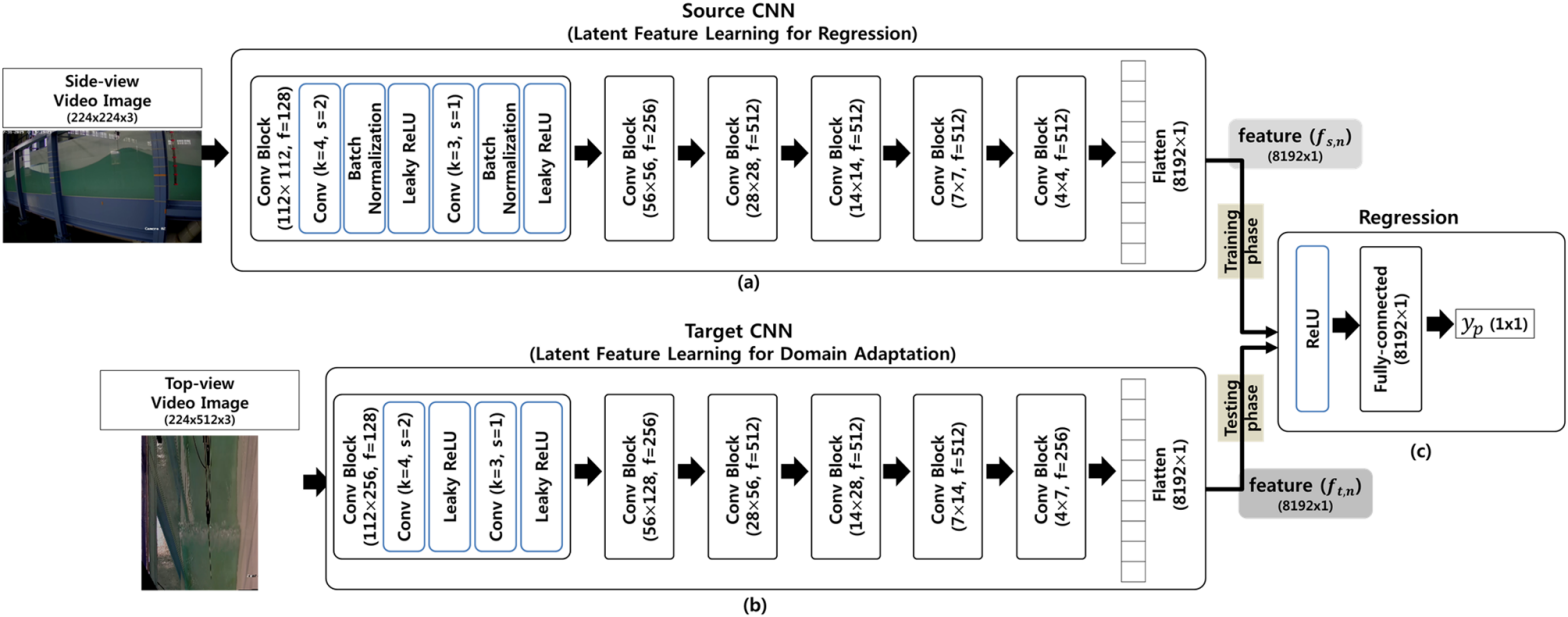
Architecture of (**a**) **Source CNN**, (**b**) **Target CNN**, and (**c**) **Regression** Network for water level estimation though domain adaptation.

In our approach, the source task is the accurate water level estimation from the side-view video images, achieved by a CNN model and *Regression* model (see *Source CNN* and *Regression*, as shown in Fig. 9). As the side-view video images contain water region information relevant to the water level, the *Source CNN* can learn meaningful latent features via convolution blocks, as described in Fig. 9, for the regression task of estimating the water level. The *Source CNN* and *Regression* models were trained in an end-to-end manner using the side-view images and water level measurement described in the subsection immediately above. The loss function for the *Source CNN* and *Regression* models are as follows:

5 $$\begin{aligned} L_{reg} = \sum _{I_{side,t} \in {{\mathbb {I}}}_{side}} ||R(S(I_{side,t}))-h_{t}||^2_2 \end{aligned}$$

where ${{\mathbb {I}}}_{side}$ is the training set of the side-view images($I_{side,t}$) for each time point (*t*) and $h_{t}$ is the water level at the corresponding time point. *S* and *R* indicate the *Source CNN* and *Regression* models, respectively. The *Source CNN* comprises six convolutional blocks. Moreover, each convolution block comprise two convolutional layers, batch normalization^18^, and Leaky ReLU activation function as described in Fig. 9a. After the conduction of the six convolution blocks, the latent features are flattened as the input of the *Regression* model. The *Regression* model is a simple fully-connected layer with ReLU activation function.

The *Target CNN* learns non-linear mapping between the top-view video images and latent features of the *Source CNN*. This deep visual domain adaptation process minimizes the source and target representation distances by iteratively minimizing the following loss function:

6 $$\begin{aligned} L_{d} = \sum _{I_{side,t} \in {{\mathbb {I}}}_{side}, I_{top,t} \in {{\mathbb {I}}}_{top}} ||S(I_{side,t}) - T(I_{top,t})||^2_2 \end{aligned}$$

where ${{\mathbb {I}}}_{top}$ is the training set of the top-view images($I_{top,t}$) for each time point (*t*) and *T* indicates the non-linear mapping of the $I_{top,t}$ to the latent feature of the source CNN with the side-view images($I_{side,t}$) via the *Target CNN*.

This joint representation learning is achieved using *L*2 loss function in Eq. (6). In the testing stage, the output latent features of the *Target CNN* are given to the *Regression* model to estimate the water level from the top-view images. In order to adapt the domain from the side-view to top-view of the wave flume video images, two independent convolutional networks (*S* and *T*) for the spatio-temporal feature extraction are implemented, as shown in Fig. 9. In the *Source CNN* (Fig. 9a), it first learns the latent feature in order to accurately measure the water levels in the side-view wave flume videos using the model for image segmentation described in the subsection immediately above. The* Target CNN* (Fig. 9b) later learns the latent feature of the top-view video images to estimate water level from the *Source CNN* feature by means of joint representation learning using *L*2 loss function in Eq. (6).

After completion of training of the model, the latent features from the top-view data are successfully adapted to the latent features from the side-view in accordance with the time signatures. The model enables the water elevation to be estimated from top-view video images and the accuracy to be evaluated by comparing the water level from the wave gauge data.

### Implementation details

The dataset used for the water level image segmentation is presented in Table 7 using side-view video images. Of the total data, the ratio of training, validation, and test data was roughly 2:1:1.

**Table 7:** Overview of data description on side view wave flume video images for semi-supervised image segmentation.

Classification	Wave type	Total video time (min.)	No. video frame	No. slice image ($200 \times 200$)	No. labeled slice image		
					(A)	(B)	(C)
Training	Regular	60	108,938	501	183	140	169
	Irregular	82	147,630	673	496	279	148
Validation	Regular	31	57,275	263	93	59	76
	Irregular	45	81,511	372	270	185	109
Test	Regular	30	54,541	251	91	50	75
	Irregular	40	73,298	335	247	162	61

The length of the time-stack images at each of the three locations, (A), (B), and (C), shown in Fig. 1a is equal to the total number of frames. Furthermore, as slice images of $220\times 220$ in size were extracted from the time-stack images, obtained from all three locations of (A), (B), and (C). In Table 7, the number of slice images represents the number of slice images at a single location. For the semi-supervised time-stack image segmentation, we acquired initial training sets by applying the validity evaluation (Supplementary Alg. 1) to all slice images. The number of the valid slice images at each location is denoted in the last three columns of Table 7.

The U-Net based deep model for semi-supervised time-stack image segmentation was trained using the Adam optimizer^19^ with batch size 30, iteration 20,000, learning rate 0.001, $\beta 1$ of 0.9, and $\beta 2$ of 0.99. In the training phase, input slice images were acquired by random extraction with sliding window ($220\times 220$ in size) in the valid regions of the time-stack image (See Supplementary Fig. 1b).

In Table 7, the total video time and the number of video frame indicates the size of training, validation, test sets for the deep visual domain adaptation models to estimate water levels from the top-view video images. Because the video images from the side and top-views are synchronized in time, the number of the top-view video frames is same as that of the side-view video frames. For the training of the Source CNN and Regression models in an end-to-end manner, we used the Adam optimizer with batch size 64, iteration 20,000, learning rate 0.0001, $\beta 1$ of 0.9, and $\beta 2$ of 0.999. The Target CNN was also trained with the same settings as the Source CNN training.

All of the experiments were conducted on a workstation equipped with a single TITAN RTX 2080Ti (11 GB), Intel i9 CPU, and 32 GB main memory. The training time depended on the size of the training data. We trained the proposed networks with the observation of the validation loss in order to avoid over-fitting and it took about 3 days of training time.

## Supplementary Information

Supplementary Information.

## References

[1] Elko N (2014). The future of nearshore processes research. AGU Fall Meeting Abstracts.

[2] Holman R, Haller MC (2013). Remote sensing of the nearshore. Annu. Rev. Mar. Sci..

[3] Vousdoukas M (2014). The role of combined laser scanning and video techniques in monitoring wave-by-wave swash zone processes. Coast. Eng..

[4] Kim J, Kim J, Kim T, Huh D, Caires S (2020). Wave-tracking in the surf zone using coastal video imagery with deep neural networks. Atmosphere.

[5] Minaee, S. *et al.* Image segmentation using deep learning: A survey. *IEEE Transactions on Pattern Analysis and Machine Intelligence* (2021). 10.1109/TPAMI.2021.3059968 33596172

[6] Ye, M. *et al.* Deep learning for person re-identification: A survey and outlook. *IEEE Transactions on Pattern Analysis and Machine Intelligence* (2021). 10.1109/TPAMI.2021.3054775 33497329

[7] Wang M, Deng W (2018). Deep visual domain adaptation: A survey. Neurocomputing.

[8] Papandreou, G., Chen, L.-C., Murphy, K. P. & Yuille, A. L. Weakly-and semi-supervised learning of a deep convolutional network for semantic image segmentation. In *Proceedings of the IEEE international conference on computer vision*, 1742–1750 (2015).

[9] Oh S-H (2018). Two-dimensional wave flume with water circulating system for controlling water level. J. Korean Soc. Coast. Ocean Eng..

[10] Andersen TL, Clavero M, Frigaard P, Losada M, Puyol J (2016). A new active absorption system and its performance to linear and non-linear waves. Coast. Eng..

[11] Foley JD (1996). Computer Graphics: Principles and Practice.

[12] Tustison, N. & Gee, J. Introducing dice, Jaccard, and other label overlap measures to itk. *Insight J.* **2** (2009).

[13] Wang Z, Xiong J, Yang Y, Li H (2017). A flexible and robust threshold selection method. IEEE Trans. Circuits Syst. Video Technol..

[14] Kim J (2020). Raindrop-aware GAN: Unsupervised learning for raindrop-contaminated coastal video enhancement. Remote Sens..

[15] Van Engelen JE, Hoos HH (2020). A survey on semi-supervised learning. Mach. Learn..

[16] Elfring, J. Image processing using opencv. Online (Feb 2018) (2013).

[17] Ronneberger, O., Fischer, P. & Brox, T. U-net: Convolutional networks for biomedical image segmentation. In *International Conference on Medical Image Computing and Computer-Assisted Intervention*, 234–241 (Springer, 2015).

[18] Ioffe, S. & Szegedy, C. Batch normalization: Accelerating deep network training by reducing internal covariate shift. arXiv preprint arXiv:1502.03167 (2015).

[19] Kingma, D. P. & Ba, J. Adam: A method for stochastic optimization. *International Conference on Learning Representations* (2014).

