# Supplementary material for: Deep visual domain adaptation and semi-supervised segmentation for understanding wave elevation using wave flume video images
Source: Sci Rep. 2021 Nov 5;11:21776. doi: 10.1038/s41598-021-01157-x (PMC8571332; doi:10.1038/s41598-021-01157-x)
Supplement: Supplementary file 1 — Supplementary Information. [file 41598_2021_1157_MOESM1_ESM.pdf]

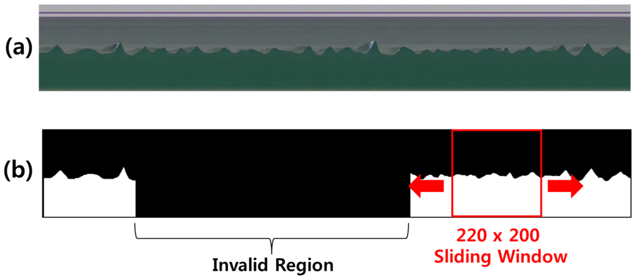

Figure 1: (**a**) Sample of a time-stack image for the video imagery in the irregular wave experiment and (**b**) result of *FloodFill* algorithm using the above time-stack image to obtain automatically labeled data for image segmentation (i.e. segmentation mask)

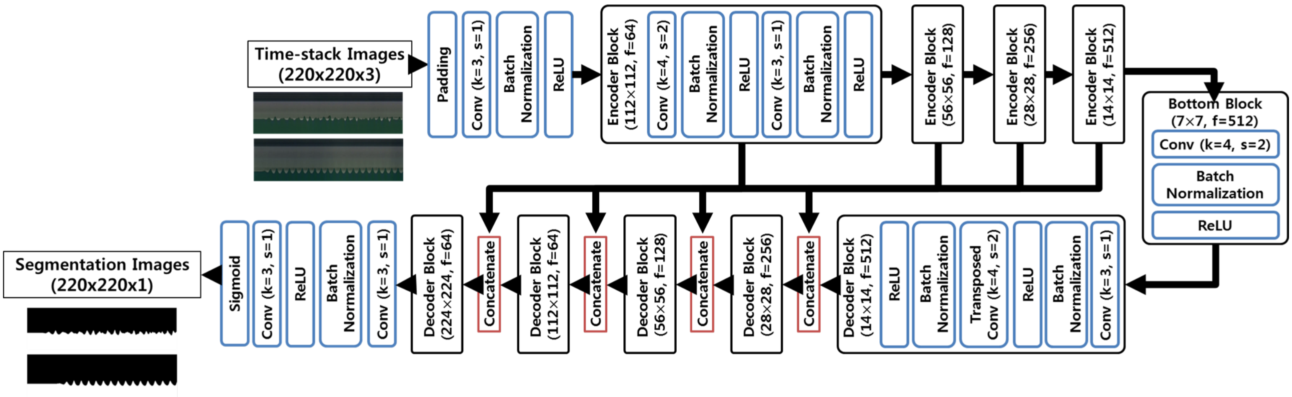

Figure 2: Architecture of U-Net for image segmentation network that only distinguishes water wave boundaries in the time-stack image of propagated water waves

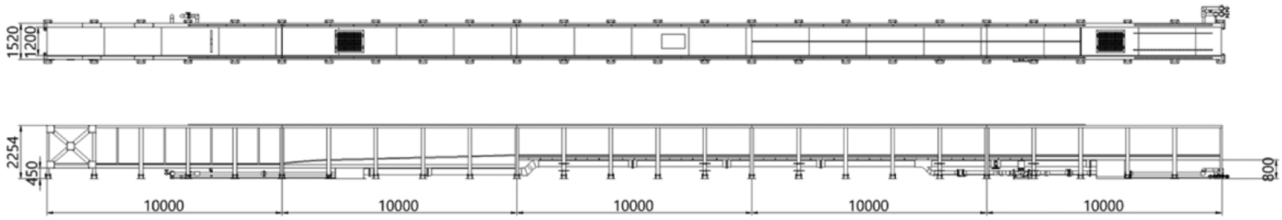

Figure 3: Plan and side views of the 2D wave flume

Algorithm 1 Is_valid(M)
: Check if the given segmentation map M is valid

Input: Segmentation map $M[1{:}H,\ 1{:}W]$ with resolution of W x H

for $h = 1$ to H **do**
  if min($M[h,\ 1{:}W]$) = max($M[h{,}1{:}W]$) **then**
    return *invalid*
  end if
end for
for $w = 1$ to W **do**
  for h 1 to H ? 1 **do**
    if $M[h,\ W]$ and $M[h{+}1,\ w]{=}0$ **then**
      (1: water, 0: background)
      return *invalid*
    end if
  end for
end for
return *valid*

Output: The validity of segmentation map M

Algorithm 2 Semi-supervised Learning of the U-Net based the time-stack image segmentation

Input: Set of slice images $\mathbb{I}_{stack} = \{I_1, I_2, ..., I_N\}$, U-Net $U(I_n;\ \phi)$, The number of iterations K

for $k = 1$ to K **do**
  $\mathbb{M} \leftarrow \emptyset$
  for $n = 1$ to N **do**
    if k = 1 **then**
      $m_n \leftarrow$ FloodFill(I_n)
    else
      $m_n \leftarrow U(I_n;\ \phi)$
    end if
    $\mathbb{M} \leftarrow \mathbb{M} \cup \{m_n\}$
  end for
  do
    for $n = 1$ to N **do**
      if Is_valid(m_n) = valid **then**
        $\phi \leftarrow \phi - \lambda \nabla_\phi\ L_{seg}(U(I_n;\ \phi), m_n)$
      end if
    end for
  while not converged
end for

Output: Optimized set of U-Net parameters ϕ

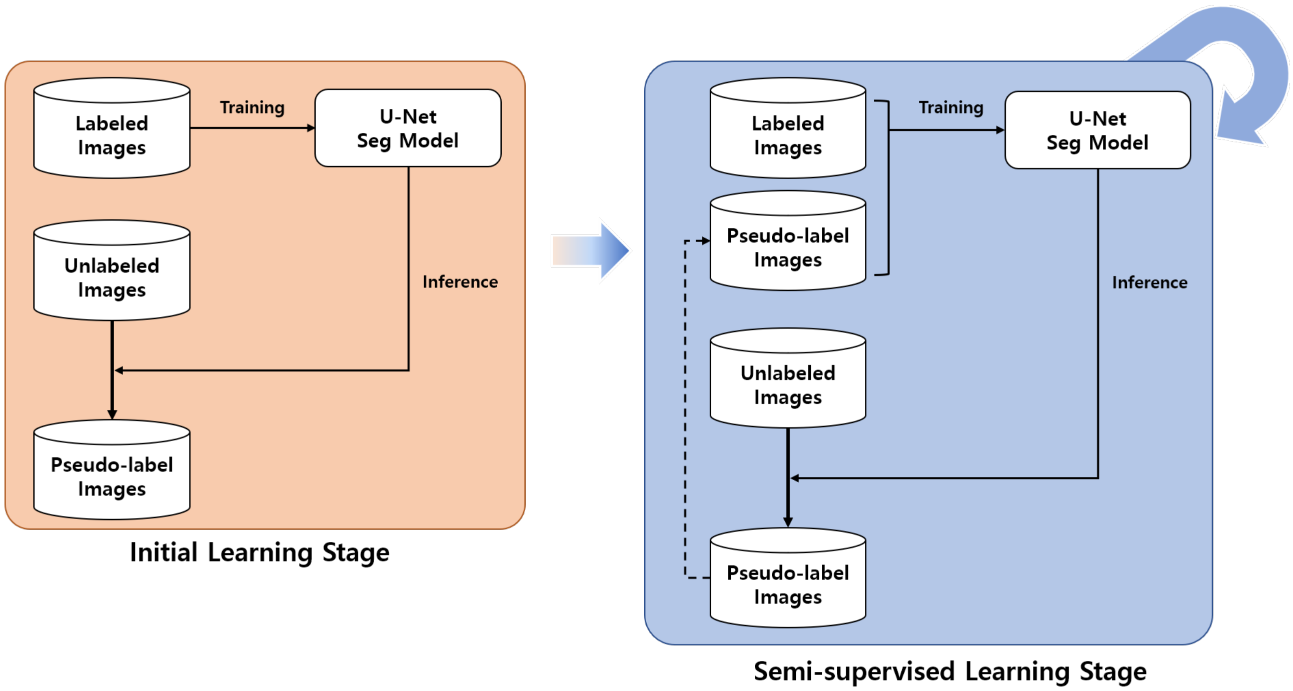

Figure 4: Flow chart of the semi-supervised learning of the U-Net for the time-stack image segmentation

Case No.	Water depth		Target Waves		Measuring time
	h_1 (cm)	h_2 (cm)	T (sec.)	H (cm)	(sec.)
1	75.4	40	1.50	14	60
2				18	
3				22	
4				26	
5			1.75	10	
6				14	
7				18	
8			.	22	
9			2.00	14	
10				18	
11				22	
12				26	
13			2.25	10	
14				14	
15				18	
16				22	
17	95.4	60	1.50	22	120
18				26	
19				30	
20				34	
21			1.75	26	
22				30	
23				34	
24				38	
25			2.00	22	
26				26	
27				30	
28				34	
29			2.25	18	
30				22	
31				26	
32				30	
33			2.50	26	
34				30	
35				34	
36				38	
37	85.4	50	1.50	30	120
38			1.75	30	
39			2.00	30	
40			2.25	26	
41			2.50	30	
42	75.4	40	1.50	26	
43			1.75	22	
44			2.00	26	
45			2.25	22	
46			2.50	22	

Table 1: Specification of experimental conditions for regular waves in the wave flume. Cells colored in yellow represent individually selected experimental cases among the test dataset to evaluate the model performance according to the wave characteristics and locations of (A), (B), and (C) in Figure 1(a)

Case No.	Water depth		Target Waves		Measuring time (sec.)
	h_1 @x=9 m (cm)	h_2 @x=30 m (cm)	T_s (sec.)	H_s (cm)	
1	85.4	50	2.00	16	800
2				20	
3				24	
4			2.50	16	925
5				20	
6				24	
7			1.50	15	375
8				20	
9			2.00	15	500
10				20	
11			2.50	15	625
12				20	

Table 2: Specification of experimental conditions for irregular waves in the wave flume. Cells colored in yellow represent individually selected experimental cases among the test dataset to evaluate the model performance according to the wave characteristics and locations of (A), (B), and (C) in Figure 1(a)

Wave Type	Case No.	R			$RMSE$ (cm)		
		(A)	(B)	(C)	(A)	(B)	(C)
Regular	20	0.98	0.97	0.97	2.75	2.34	2.49
	24	0.95	0.99	0.99	3.60	1.42	2.51
	32	0.94	0.96	0.99	3.71	2.77	2.30
	36	0.97	0.98	0.98	3.39	2.23	3.14
	40	0.94	0.96	0.99	3.47	2.59	2.11
	45	0.96	0.97	0.99	2.71	2.14	1.78
Irregular	4	0.82	0.91	0.98	2.63	1.65	1.02
	8	0.84	0.84	0.98	2.17	2.19	1.03
	12	0.83	0.91	0.99	2.63	1.99	1.19

Table 3: Performance of measured water elevation from side-view video images through seimi-supervised learning on some experimental cases to examine the performance according to wave characteristics and locations of (A), (B), and (C) among test dataset

Wave Type	Case No.	R			$RMSE$ (cm)		
		(A)	(B)	(C)	(A)	(B)	(C)
Regular	20	0.91	0.98	0.98	5.27	2.64	3.93
	24	0.81	0.89	0.91	7.81	7.10	6.72
	32	0.95	0.97	0.99	4.58	2.80	3.10
	36	0.97	0.98	0.98	4.34	3.20	4.22
	40	0.96	0.97	0.98	3.30	3.06	2.89
	45	0.93	0.96	0.98	3.83	2.71	2.68
Irregular	4	0.85	0.91	0.96	2.15	1.77	1.30
	8	0.65	0.84	0.83	3.23	2.96	2.03
	12	0.83	0.91	0.96	2.87	2.20	1.71

Table 4: Performance of estimated water elevation from top-view video images through deep visual domain adaptation on some experimental cases to examine the performance according to wave characteristics and locations of (A), (B), and (C) among test dataset
